# A Comparative Study in Learning Curves of Two Different Intracorporeal Knot Tying Techniques

**DOI:** 10.1155/2016/3059434

**Published:** 2016-02-28

**Authors:** Manuneethimaran Thiyagarajan, Chandru Ravindrakumar

**Affiliations:** General Surgery Department, Sri Ramachandra Medical University, Chennai, India

## Abstract

*Objectives*. In our study we are aiming to analyse the learning curves in our surgical trainees by using two standard methods of intracorporeal knot tying.* Material and Method*. Two randomized groups of trainees are trained with two different intracorporeal knot tying techniques (loop and winding) by single surgeon for eight sessions. In each session participants were allowed to make as many numbers of knots in thirty minutes. The duration for each set of knots and the number of knots for each session were calculated. At the end each session, participants were asked about their frustration level, difficulty in making knot, and dexterity.* Results*. In winding method the number of knots tied was increasing significantly in each session with less frustration and less difficulty level.* Discussion*. The suturing and knotting skill improved in every session in both groups. But group B (winding method) trainees made significantly higher number of knots and they took less time for each set of knots than group A (loop method). Although both knotting methods are standard methods, the learning curve is better in loop method.* Conclusion*. The winding method of knotting is simpler and easier to perform, especially for the surgeons who have limited laparoscopic experience.

## 1. Introduction

A surgeon needs to improve his laparoscopic skills to overcome the difficulties in laparoscopy, which can be achieved through multiple and repetitive sessions in an alternative training method rather than getting trained in the patients directly. All must master the difficulties such as loss of depth perception, fulcrum effect of abdominal wall, limited motion freedom, and manipulation of tissues. This can be made easy by practicing in any of the training materials such as surgical simulators, virtual simulating trainers, and box-trainers. But the haptic feedback is best learnt in a box-trainer [1], which is an essential thing for learning the skills.

One of the most difficult tasks in laparoscopic surgery is intracorporeal knot tying and suturing technique. The fundamental elements of knot tying are its safety, easiness, rapidity of execution and tightness, and maintaining the knot. It has been an obstacle to all learners as it requires a lot of technical movement in a limited space. The extracorporeal knot tying is one of the alternate methods to overcome this difficulty [2].

When angle between the working instruments is narrow, the ligation and suturing are very difficult to perform. A side winding intracorporeal suturing can overcome this difficulty [3]. Most importantly geometric factors of endoscopic reconstruction, such as optimal distances between the working trocars, length of instruments, and angles between the instruments and the object will make lot of difference in suturing. Optimal geometry for intracorporeal suturing can be achieved by creating an isosceles triangle between the instruments with an angle between 25° and 45° and an angle <55° between the instruments and the horizontal line. These data should be considered when planning reconstructive laparoscopic procedure [4]. To make secured intracorporeal knot, surgeons knot is the ideal knotting method which consists of 2 × 1 configuration. The main aim of intracorporeal knot tying is to control suture tension and to create square knot in secured method. For basic laparoscopic surgery like appendectomy, a pretied loop can be used [5]. For single instrument knotting* Dowais Tie* technique [6] can be used. In a conventional knotting method with two instruments, the* loop* is made with one hand instrument and other hand instrument enters into it to catch the tail end. An alternative method is by holing the suture half centimeter distal to the needle with one hand instrument and to rotate to make* winds* [7, 8] while the other hand instrument maintains the winds to apply the knot. There are many studies explaining about the different types of intracorporeal knot and its learning curves. But there is no information available about the best method of knot tying for teaching the surgical trainees in the box-trainer. In our study we are aiming to analyse the learning curves in our surgical trainees by using two different intracorporeal knot tying methods.

## 2. Materials and Methods

Twenty postgraduate students studying in the Department of General Surgery at Sri Ramachandra Medical College and Research Institute were included in this study. They participated on a voluntary basis. Students with prior experience in laparoscopic suturing, suturing in box-trainer, and suturing in simulators were excluded. All participants were right handed and between 24 to 35 years. Participants were divided into two groups: A and B. They were randomized by using the website http://www.randomization.com/. There were ten participants for each group. Group A was asked to follow loop method of knot tying and group B was asked to follow winding method of knot tying.

Laparoscopic box-trainer was used. Two needle drivers were used for each participant. A ten-centimeter vicryl suture 2-0 (V62H-Ethicon) material was given to each participant for every set of knots (2 × 1 × 1). Handling of instrument with manipulation of button and handling of needle driver were allowed five minutes before starting the task. Suturing pad was used to anchor the suture material while applying the knot.

Prior to start with training, each participant was given explanation about the surgical technique with a video and live demonstration by a single examiner. Same examiner coached all participants throughout the entire study. Proper bite has to be taken in the suture pad at the premarked area. After pulling the needle from the suture pad 2 × 1 × 1 configuration knots were made during the training, the time taken for each set of knots was calculated, and the examiner corrected the participants whenever necessary to make the knot in a standardized manner.

### 2.1. Knot Tying Methods

#### 2.1.1. Group A (Loop Method)

**Figure 1:**
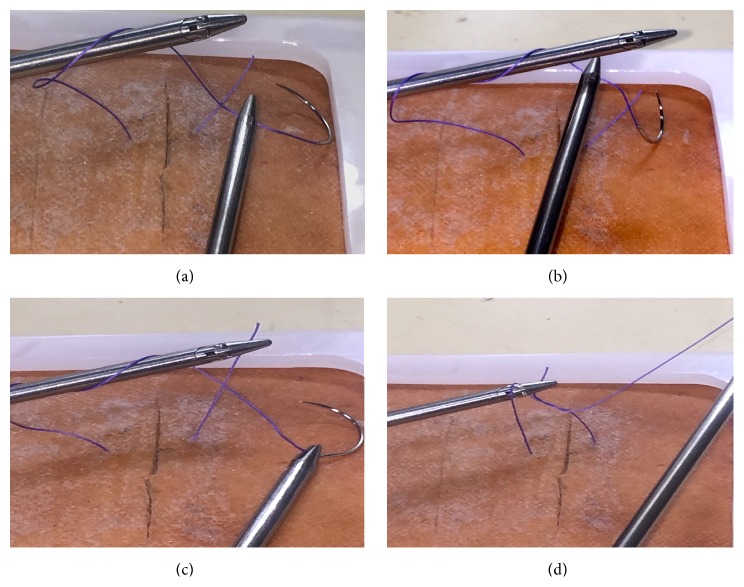
(a) (Step 1) Starting the loop of suture by right hand needle driver over the left hand needle driver. (b) (Step 2) Making two loops over the left hand needle driver. (c) (Step 3) The left hand needle driver catching the tail end of the suture through the loops. (d) (Step 4) First throw of knot completed by pulling the tail end of suture through the loop.

After delivering needle from suture pad the right hand needle driver will make two loops (double forward) over the left hand needle driver and left hand needle driver will catch the tail of the suture and squaring of knot must be done. This will be followed with single reverse loop and one forward loop (Figure 1).

#### 2.1.2. Group B (Wind Method)

**Figure 2:**
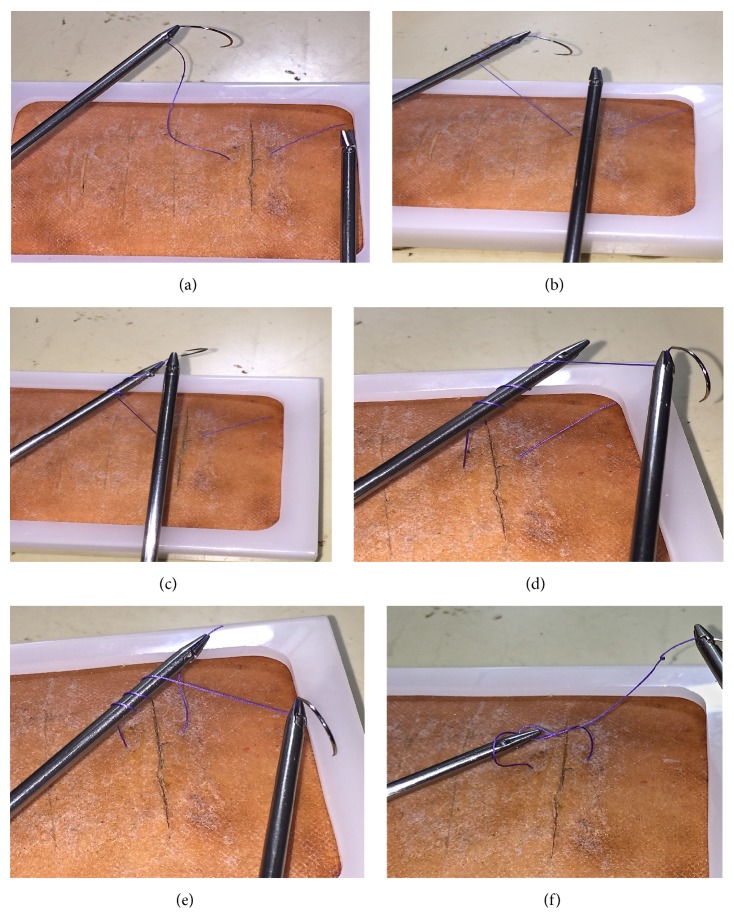
(a) (Step 1) Holding the suture material near the needle by left hand needle driver. (b) (Step 2) Winding of suture around the left hand needle driver. (c) (Step 3) Right hand needle driver receiving the needle from the left hand needle driver. (d) (Step 4) Right hand needle driver maintaining the wind on left needle driver. (e) (Step 5) Left hand needle driver catching the tail end of suture. (f) (Step 6) First throw of knot completed by pulling the tail end of suture.

After delivering the needle, the driver will drop the needle and the left needle driver will hold the suture half centimeter distal to the needle. Then the left needle driver has to rotate to wind the thread around its axis. The right needle driver has to then receive the needle once adequate winding was done followed by left needle driver to catch the tail end to make a square knot. The second throw will be done by reverse wind and last part will be done by forward wind (Figure 2).

Totally thirty-minute time was given for each participant for each session. Participants were allowed to make as many numbers of knots as possible for each session. Time calculation was done for each set of knots. The number of knots for each session was also calculated. At the end each session's participants were asked for information about frustration level (ranging from 1 to 5, 1 being the least and 5 being the maximum), difficulty in making knot (ranging from 1 to 5, 1 being the least and 5 being the maximum), and dexterity (ranging from 1 to 5, 1 being the least and 5 being the highest).

### 2.2. Statistical Methods

The collected data was analysed with SPSS 16.0 version software. To describe the data descriptive statistics, mean and standard deviation were used. To find the significant difference between the bivariate samples in paired groups (between sessions 1 to 8) Wilcoxon signed rank test was used and for independent groups (loop and wind) Mann-Whitney *U* test was used. For the repeated measures (from sessions 1 to 8) the Friedman test was used. In all the above statistical tools the probability value <0.05 is considered as significant level.

## 3. Results

### 3.1. Number of Knots

**Figure 3:**
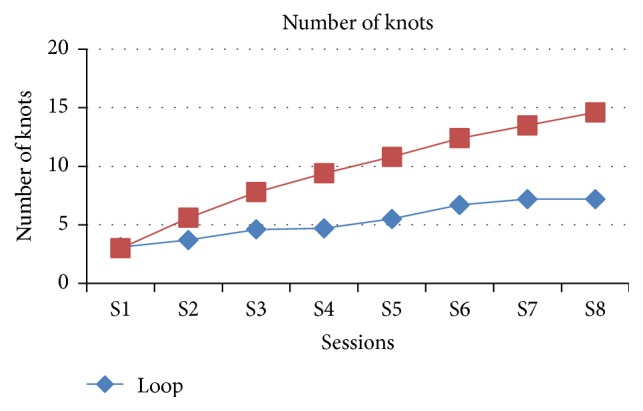
Graphical representation of both groups in view of number of knots in each session.

The comparison of number of knots along the sessions between the loop and winding methods showed that as the sessions were increasing the number of knots tied was increasing in the winding method compared with the loop method which is statistically significant except the first session, *P* = 0.853 > 0.05. The maximum number of knots made along the session in loop technique was seven and it shows a steady increase only from the sixth session, whereas in the winding technique there was a consistent increase in the number of knots from the first session to eighth session and it reached a maximum of fifteen knots at the end of the eighth session itself (Table 1, Figure 3).

### 3.2. Average Time Taken

**Figure 4:**
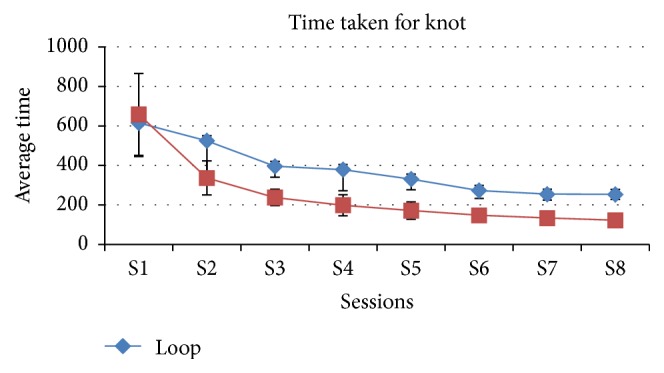
Graphical representation of both groups in view of average time in seconds for single set of knots in each session.

The comparison of average time taken along the sessions between the loop and winding method shows that as the sessions were increasing the average time decreases in the winding method compared with the loop method which is statistically significant except the first session, *P* = 0.436 > 0.05 (Table 2, Figure 4).

### 3.3. Frustration Level

**Figure 5:**
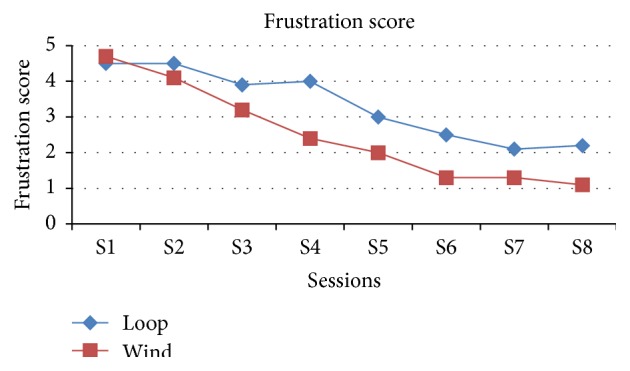
Graphical representation of both groups in view of frustration in the end of each session.

The comparison of frustration level along the sessions between the loop and winding method shows that as the sessions were increasing the frustration score is decreasing in the winding method and becomes steadily 1 from sixth session onwards compared with the loop method which is 2 till the end of the eighth session and which is statistically significant except the first three sessions (Table 3, Figure 5).

### 3.4. Difficulty Level

**Figure 6:**
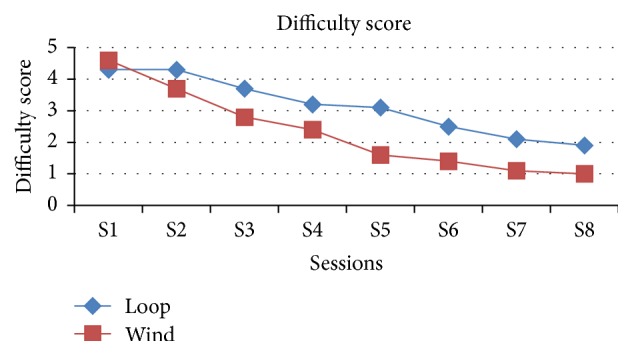
Graphical representation of both groups in view of difficulty in making knot in the end of each session.

The comparison of difficulty level along the sessions between the loop and winding method shows that as the sessions were increasing the difficulty score is decreasing in the winding method and becomes steadily 1 from sixth session onwards compared with the loop method which is 2 till the end of the eighth session and which is statistically significant except the first three sessions (Table 4, Figure 6).

### 3.5. Dexterity

**Figure 7:**
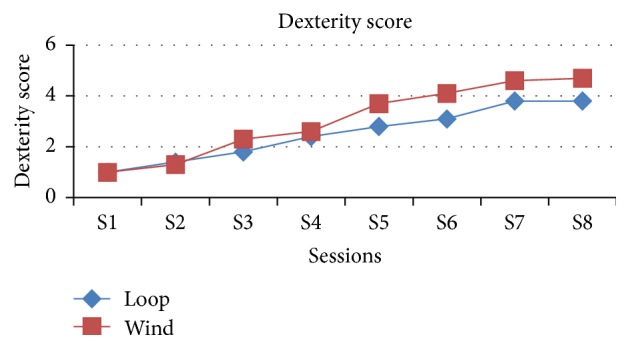
Graphical representation of both groups in view of dexterity of complete procedure in the end of each session.

The comparison of dexterity along the sessions between the loop and winding method shows that as the sessions were increasing the dexterity score is increasing in the winding method and becomes steadily 5 on seventh session itself compared with the loop method which is 4 till the end of the eighth session and which is statistically significant except the first three sessions (Table 5, Figure 7).

### 3.6. Comparison of Session 8 with All Other Sessions in Both Loop Method and Winding Method

In winding method, while comparing the average time for suturing in S8 with other sessions, there is significant improvement in every session (*P* value < 0.01). It means average time taken for knot tying decreases significantly from S1 to S8 (Table 6).

In loop method, while doing the same type of comparison, there is significant improvement seen only up to S5. In S6 and S7 no significant improvement was seen. It means that there is no significant reduction in average time of knot tying from session 6 to session 8.

## 4. Discussion

It is well established that minimally invasive surgical skill must be acquired by proper training in box-trainers, rather than directly performing in a clinical setting to ensure patient safety. Training method has been changed currently like “see one, do one, and teach one.” But in laparoscopy this may not be applicable since there is a long learning curve. The haptic feedback in box-trainer will definitely improve the surgeon's skill. To avoid the injury to the abdominal organs during surgery the moving part of instrument must be in the optic field [9]. This training can be achieved in box-trainer. The working port placement must be either side of camera to make adequate triangulation, which will make knot tying easier. In box-trainer the port placement will be ideal for making triangulation. For suturing and knotting standard technique must be followed. After taking bite from tissue surgeon must pull the suture to make tail end shorter as 2 cm and he should position it before making knot. This will make grasping the tail end after making wind or loop easy [10]. To make knot in the tissue there are many knots tying methods available. For single port users extracorporeal knot tying method may be useful. To make knot tying as strong as open surgery at least two hands instruments (right and left) must be used [11, 12].

There is no study to compare the different suturing techniques in trainees. Zhou et al. work was a comparative study of suturing practice with haptic feedback and without haptic feedback. In this study result showed haptic feedback may not be warranted in laparoscopic surgical trainers at all stages of training [1]. In our study the training was given in the box-trainer. Haptic feedback of box-trainer definitely improved the suturing skills among our postgraduate students. From session 1 to session 8 there is lot of improvement in speed and dexterity in both groups. This shows the learning curve of both groups going up by our box-training. The suturing and knotting skill improved in every session in both groups. In comparison, group B (winding method) students made significantly higher number of knots and they took less time for each knot than group A (loop method) in all sessions except session 1. The feedback results of the candidates show no significant difference in view of frustration, difficulty, and dexterity from S1 to S3. On the other hand from S4 to S8, the difficulty and frustration significantly reduced and dexterity improved in group B (winding method) compared with group A (loop method).

While comparing each session with last session, in Group A (loop method) there is statistically significant improvement from S1 to S5, but from S6 to S8 there is no significant improvement in knot tying. In contrast there is significant improvement in winding method completely from S1 to S8.

All the above results show there is significant improvement in the learning curve in every session of training in both groups. Although both knotting methods are standard methods to apply in patients, the winding method of knotting will be simple and easy for the beginners to learn. In winding method of suturing the triangulation of working instrument is not completely required but in loop method of suturing the triangulation and adequate distance between instruments are required. Jagad explained the advantage of this winding method of suturing. It explains that winding method of suturing is more simple and easy to perform, especially for those who have limited experience in intracorporeal suturing and knot tying. It is easy to perform in narrow space. No special instrument is required to perform knot tying with this technique [8]. Even in our study frustration level and difficulty level in winding method are less comparing with loop method and reason could be the same as that mentioned by Jagad. The ergonomics of suturing is very important which will make surgery easier and faster. In winding method of suturing the ergonomic is improved and that is the reason why trainees can make more number of sutures with less difficulty. Rassweiler et al. explain that an ergonomic chair will improve the suturing in laparoscopic surgery [13]. In our study results show the winding method suturing is easier than loop method suturing. So This method of suturing can improve ergonomics and can make reconstructive laparoscopic surgeries easier. Murphy explained about advance modern surgical techniques of endoscopic knot tying, a new appreciation of knot tying theory, and application of these new techniques in many fields of minimally invasive surgery. In our study two standard methods of suturing techniques were compared which may give useful information to identify the best method of suturing technique in various types of minimally invasive surgeries.

## 5. Conclusion

Laparoscopic suturing and knotting are difficult skills to develop especially in new learners. Even though there are many methods of intracorporeal suturing available, two best methods of suturing techniques were compared in view of learning curves. While comparing with loop method of knotting, the winding method of knotting is simpler and easier to perform, especially for the surgeons who have limited experience in intracorporeal suturing and knot tying. The winding method essay for the learners probably does not require much triangulation while making knot.

## Figures and Tables

**Table 1:** Comparison of average number of knots was done between groups A and B (decimal number converted to whole number).

Comparison of number of knots along the sessions between loop & winding method								
	S1	S2	S3	S4	S5	S6	S7	S8
Loop	3	4	5	5	6	7	7	7
Wind	3	6	8	9	11	12	14	15
*Z*-value	0.24	3.14	3.77	3.58	3.78	3.81	3.83	3.87
*P* value	0.853^#^	0.0001^*∗∗*^	0.0001^*∗∗*^	0.0001^*∗∗*^	0.0001^*∗∗*^	0.0001^*∗∗*^	0.0001^*∗∗*^	0.0001^*∗∗*^

^#^Not sig. at *P* ≤ 0.05  and  ^*∗∗*^highly sig. at *P* ≤ 0.01 level.

**Table 2:** Comparison of average time for one set of knots (2 × 1 × 1) in every session was done between groups A and B.

Comparison of average time along the sessions between loop & winding method								
	S1	S2	S3	S4	S5	S6	S7	S8
Loop	615.7	525.0	395.9	378.3	330.4	272.8	254.2	253.2
Wind	658.1	336.5	237.6	198.2	171.5	147.0	133.5	122.3
*Z*-value	0.795	3.176	3.704	3.593	3.78	3.781	3.781	3.784
*P* value	0.436^#^	0.0001^*∗∗*^	0.0001^*∗∗*^	0.0001^*∗∗*^	0.0001^*∗∗*^	0.0001^*∗∗*^	0.0001^*∗∗*^	0.0001^*∗∗*^

^#^Not sig. at *P* ≤ 0.05  and  ^*∗∗*^highly sig. at *P* ≤ 0.01 level.

**Table 3:** Comparison of frustration level at the end of every session was done between groups A and B (from 1, least frustration, to 5, maximum frustration).

Comparison of frustration score along the sessions between loop & winding method								
	S1	S2	S3	S4	S5	S6	S7	S8
Loop	5	5	4	4	3	3	2	2
Wind	5	4	3	2	2	1	1	1
*Z*-value	1.129	1.258	1.802	3.297	2.457	3.17	2.675	3.442
*P* value	0.353^#^	0.28^#^	0.089^#^	0.001^*∗∗*^	0.019^*∗∗*^	0.002^*∗∗*^	0.011^*∗∗*^	0.001^*∗∗*^

^#^Not sig. at *P* ≤ 0.05  and  ^*∗∗*^highly sig. at *P* ≤ 0.01 level.

**Table 4:** Comparison of difficulty level at the end of every session was done between groups A and B (from 1, least difficult level, to 5, highest difficult level).

Comparison of difficulty score along the sessions between loop & winding method								
	S1	S2	S3	S4	S5	S6	S7	S8
Loop	4	4	4	3	3	3	2	2
Wind	5	4	3	2	2	1	1	1
*Z*-value	1.314	1.849	1.961	2.737	3.071	3.006	3.88	3.943
*P* value	0.280^#^	0.089^#^	0.063^#^	0.011^*∗∗*^	0.002^*∗∗*^	0.003^*∗∗*^	0.0001^*∗∗*^	0.0001^*∗∗*^

^#^Not sig. at *P* < 0.05  and  ^*∗∗*^highly sig. at *P* < 0.01 level.

**Table 5:** Comparison of dexterity (1–5) in the end of session was done between groups A and B.

Comparison of dexterity score along the sessions between loop & winding method								
	S1	S2	S3	S4	S5	S6	S7	S8
Loop	1	1	2	2	3	3	4	4
Wind	1	1	2	3	4	4	5	5
*Z*-value	0	0.457	1.832	0.602	2.669	2.523	2.437	2.466
*P* value	0.280^#^	0.089^#^	0.063^#^	0.011^*∗∗*^	0.002^*∗∗*^	0.003^*∗∗*^	0.0001^*∗∗*^	0.0001^*∗∗*^

^#^Not sig. at *P* < 0.05  and  ^*∗∗*^highly sig. at *P* < 0.01 level.

**Table 6:** Comparison of statistical significance for every session in both groups.

*P* values									
	Session 1	Session 8	S1 versus S8	S2 versus S8	S3 versus S8	S4 versus S8	S5 versus S8	S6 versus S8	S7 versus S8
LOOP	615.7 ± 170	253.1 ± 25	0.005^*∗∗*^	0.005^*∗∗*^	0.005^*∗∗*^	0.005^*∗∗*^	0.005^*∗∗*^	0.025^*∗*^	0.159^#^
WIND	658.3 ± 207	122.3 ± 20	0.005^*∗∗*^	0.005^*∗∗*^	0.005^*∗∗*^	0.005^*∗∗*^	0.005^*∗∗*^	0.005^*∗∗*^	0.007^*∗∗*^

^#^Not sig. at *P* ≤ 0.05, ^*∗*^sig. at *P* < 0.05 level, and  ^*∗∗*^highly sig. at *P* < 0.01 level.
