# Corrigendum to “A Comparative Study in Learning Curves of Two Different Intracorporeal Knot Tying Techniques”

**DOI:** 10.1155/2018/7646831

**Published:** 2018-05-20

**Authors:** Manuneethimaran Thiyagarajan, Chandru Ravindrakumar

**Affiliations:** General Surgery Department, Sri Ramachandra Medical University, Chennai, India

In the article titled “A Comparative Study in Learning Curves of Two Different Intracorporeal Knot Tying Techniques” [1], Dr. Manuneethimaran Thiyagarajan was incorrectly listed as the corresponding author. The corresponding author is Dr. Chandru Ravindrakumar.
